# Video-Assisted Informed Consent for Cataract Surgery: A Randomized Controlled Trial

**DOI:** 10.1155/2017/9593631

**Published:** 2017-01-16

**Authors:** Yuehong Zhang, Xiangcai Ruan, Haoying Tang, Weizhong Yang, Zhuanhua Xian, Min Lu

**Affiliations:** ^1^Department of Ophthalmology, Guangzhou First People's Hospital, Guangzhou Medical University, Guangzhou, China; ^2^Departments of Anesthesia and Pain Medicine, Guangzhou First People's Hospital, Guangzhou Medical University, Guangzhou, China; ^3^Department of Ophthalmology, Sanshui District People's Hospital of Foshan Affiliated to Guangdong Medical College, Guangdong, China

## Abstract

*Purpose*. To investigate whether adding video assistance to traditional verbal informed consent advisement improved satisfaction among cataract surgery patients.* Methods*. This trial enrolled 80 Chinese patients with age-related cataracts scheduled to undergo unilateral phacoemulsification surgery. Patients were randomized into two groups: the video group watched video explaining cataract-related consent information and rewatched specific segments of the video at their own discretion, before receiving traditional verbal consent advisement; the control group did not watch the video. Outcomes included patient satisfaction, refusal to consent, time to complete the consent process, and comprehension measured by a ten-item questionnaire.* Results*. All 80 enrolled patients signed informed consent forms. Compared with the control group, members of the video group exhibited greater satisfaction (65% versus 86%, *p* = 0.035) and required less time to complete the consent process (12.3 ± 6.7 min versus 5.6 ± 5.4 min, *p* < 0.001), while also evincing levels of comprehension commensurate with those reported for patients who did not watch the video (accuracy rate, 77.5% versus 80.2%, *p* = 0.386).* Conclusion*. The video-assisted informed consent process had a positive impact on patients' cataract surgery experiences. Additional research is needed to optimize patients' comprehension of the video.

## 1. Introduction

Informed consent is a fundamental component of modern medicine and indispensable in the practice of medicine. Effective informed consent advisement not only helps patients select treatment options by receiving adequate counseling about the indications, risks, benefits, and alternatives of medical procedures but also lowers the risk of malpractice litigation [1–3].

Traditionally, the preoperative consent procedure for cataract surgery, one of the most frequently performed eye operations, begins with a discussion between the ophthalmologist and the patient and ends with the patient signing a document confirming consent. However, the effectiveness of this traditional consent discussion has been called into question in the past because of uncertainties about the extent to which the physicians conveyed and level of understanding of the patients [4–6]. In the case of cataract surgery specifically, the process may be flawed because the patient cohort for such procedures overwhelmingly comprises the elderly and infirm [5, 7]. Moreover, a busy ophthalmological surgeon must usually perform many elective eye surgeries each day, and the informed consent processes for these operations can vary greatly.

Various media, including informational videos, have been demonstrated to improve patient comprehension in a variety of clinical settings [3, 8]. In two US eye clinics, a video presentation produced by the American Academy of Ophthalmology titled “Understanding Cataract Surgery” was found to increase patient comprehension and recall of consent information [7, 9]. Still, contradictory results have been found in emergency and pediatric settings [10, 11]. Patient satisfaction is paramount in any practice, but the effects of informational video use on patient experience in regard to cataract surgery remain largely unexamined. In this study an informational video was made and shown to a sampling of cataract surgery candidates to assess whether it improved their patient satisfaction beyond what is typically seen with traditional verbal informed consent advisement for cataract surgery.

## 2. Materials and Methods

### 2.1. Subjects

This prospective, randomized study took place at two university hospitals: Guangzhou First People's Hospital, an affiliate of Guangzhou Medical University, and Sanshui District People's Hospital of Foshan, an affiliate of Guangdong Medical College, Guangdong, China; the study was conducted in accordance with the Helsinki Declaration and was approved by each facility's institutional review board. Its registration number is NCT02185807 in the Clinicaltrials registry.

Age-related cataract patients scheduled for their first elective cataract surgeries between July 2014 and March 2015 were recruited for this study. All the patients invited to participate in the study were deemed capable of informed consent and capable of watching a video (i.e., they had a best-corrected visual acuity [BCVA] of more than 1.0 [LogMAR chart] for either eye). Patients were excluded from the sampling if any of the following was true: (1) the patient had previously undergone cataract surgery; (2) the patient was unable to watch the video and complete the self-administered questionnaire; (3) the patient was currently suffering complications from complex eye diseases, such as uveitis or retinal detachment; (4) the patient's language was not Mandarin or Cantonese.

### 2.2. Study Procedure

Eligible patients who agreed to participate in the present study were randomly assigned to two groups and completed one of two informed consent processes for cataract surgery separately. The members of the video group watched the informed consent video, received conventional verbal advisement, and read consent documents; members of the control group only received conventional verbal advisement and read consent documents. Treatment allocations were created, stratified, and balanced by a research member otherwise not involved in the study using a computer-generated randomization table. They were sequentially sealed in opaque envelopes and had no external labeling. In each center, only one person, who was neither a surgeon nor physician, had access to the envelopes, and surgeons were informed of each surgery one working day before it was to take place.

Based on the American Academy of Ophthalmology's nine-minute educational DVD “Understanding Cataract Surgery” [12] and several frequently asked questions collected from age-related cataract patients, a bilingual educational video about informed consent for cataract surgery was developed. The total runtime of the Mandarin version was six minutes, 16 seconds, and that of the Cantonese version was six minutes, 11 seconds. The DVD introduced the cataract surgery procedure and explained its risks, benefits, and alternatives with the help of an “eyeball phantom” and several visual teaching aids; it also made use of animation and music. Images, photographs, or expressions that might cause discomfort for patients were not included in the presentation. The script was written in Mandarin and Cantonese by an expert in ophthalmological patient-information needs and an ophthalmologist with standard Mandarin and Cantonese pronunciation served as the narrator.

The two versions of the video were shown simultaneously in separate rooms. Patients in the video group chose which version to see according to their language preference. They were allowed to watch and rewatch specific segments of the video without having to rewatch it in its entirety at their own discretion. They then discussed their medical options with their physician and were given consent forms to read. Patients in the control group were given the exact same information by their ophthalmologists and were given the same consent forms to read. Once patients reported adequately understanding the surgical procedure and its risks, benefits, and reasonable alternatives as well as the uncertainties endemic to each alternative, they were asked to sign the informed consent form.

To assess the effectiveness of the two informed consent processes, this study employed a self-administered 10-item questionnaire about information related to cataract surgery and its risks. Every patient was invited to complete it right after going through the informed consent process (see the following list).

*Questionnaire about the Benefits and Risks of Cataract Surgery*

1. Is it true that surgery is the most effective treatment for cataracts? Y/N
2. Is it true that your visual acuity will definitely improve after cataract surgery? Y/N
3. Does your postoperative visual acuity necessarily improve if you have an unhealthy fundus? Y/N
4. Is it true that diabetic patients cannot undergo cataract surgery? Y/N
5. Will an intraocular lens be needed to improve your postoperative visual acuity? Y/N
6. Can severe complications from cataract surgery cause severe outcomes? Y/N
7. Is advanced age a risk factor for developing cataracts? Y/N
8. Is it true that you should consider cataract surgery only when you lose your vision completely? Y/N
9. Are eye drops an alternative to cataract surgery? Y/N
10. Can cataract surgery also make your eyes uncomfortable, although it will allow you to see more clearly? Y/N.

In each center, a researcher who was masked to the grouping process and familiar with both Mandarin and Cantonese recorded the number of correct responses to the 10 questions for each group. Patients also reported their date of birth, preferred language, highest level of education, and frequency of computer use. Education level was categorized as illiterate, primary education, or secondary/higher education. Computer use was reported as rare/never or often. An evaluation of patient satisfaction with the informed consent process was performed using another self-administered questionnaire composed of four items: physician's attitude, adequate information, adequate time to receive the information, and overall satisfaction with the informed consent process. Each item was rated as “excellent,” “good,” “fair,” or “poor.” Literate patients completed the questionnaires themselves. Researchers read the questions to those who were illiterate and recorded their answers.

The time spent obtaining signed informed consent forms by physicians was also measured. In the case of the control group, the measured time included the time needed to explain the informed consent information and answer patients' questions prior to their signing. Similarly, the times recorded for the video group included the time spent on question-and-answer sessions, but the time required to watch the video was not included in the present study's measurements. These patient procedures were all completed one day prior to surgery.

### 2.3. Statistical Analysis

The sample size was based on previous experience showing that 50% of the patients who underwent cataract surgery were satisfied with the consent process. For purposes of analysis, the four-point ordinal-satisfaction scale was dichotomized into two categories:* satisfied* (for those who gave “good” and “excellent” ratings) and* dissatisfied* (for those who gave “poor” and “fair” ratings). To detect a 30% difference in patient satisfaction, it was calculated that each group had to include at least 36 patients in order to achieve an 80% power (*α* 5%). Assuming a 10% nonresponse rate, the final sample size was set at 80, with 40 patients in each group. Categorical variables underwent either a chi-square analysis or Fisher's Exact Test; continuous measurements were tested using either Student's *t*-test or ANOVA (as appropriate). Subanalysis was performed for the influence of age, gender, preferred language, level of education, and frequency of computer use. Continuous data are presented as mean and standard deviation (SD), whereas categorical data are presented as the number of patients and percentages. Data analyses were performed using* Statistical Package for the Social Sciences*, version 16 (SPSS Inc., Chicago, IL, USA).

## 3. Results

**Figure 1:**
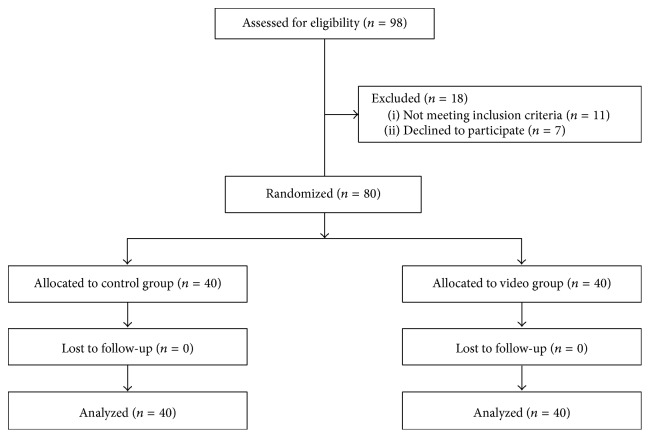
Patient flow diagram.

Figure 1 shows patient flow throughout the study. A total of 98 patients from the two sites were evaluated for study inclusion; of these, seven declined and 11 were excluded. Problems that resulted in patient exclusion included hearing problems (2), blindness (6), and patient immobility (3).  Table 1 lists the demographic characteristics of the enrolled patients. Overall, the characteristics of both groups were comparable.

All 80 patients signed an informed consent form and agreed to answer a questionnaire about the benefits and risks of their elected surgery. As expected, overall patient satisfaction for the combined groups was 75%, but it was significantly higher in the video group (86% versus 65%, *p* = 0.035, Table 2). The amount of time spent by an ophthalmologist in completing the consent process was less in the video group (5.6 ± 5.4 min versus 12.3 ± 6.7 min,* p* < 0.001). However, the number of correct responses to the questionnaire remained constant for both groups (321 correct answers in the video group versus 310 in the control group, with accuracy rates of 80.2% versus 77.5%, *p* = 0.386). Subanalysis also showed no significant differences in the number of correct responses between the different age groups, the different education groups, the gender groups, or the computer use groups (Table 3).

## 4. Discussion

The present study was conducted to assess the impact of use of video information in the consent process and assess the impact on patient satisfaction, comprehension, and time spent with ophthalmologist as part of the consent process. It did so by showing an informational video prior to traditional verbal advisement, and its main finding was showing that the video improved patient satisfaction. Furthermore, though it did not improve patient comprehension, the informational video decreased the amount of time the participating ophthalmologists spent on the consent process.

Adding an informational video to traditional verbal informed consent advisement is becoming increasingly popular, but it has also, to some extent, been subject to scrutiny. The improvement in patient satisfaction for those who viewed the video in this study is consistent with previous research on the role of videos in decision-making and consent for various surgical procedures, including gastric banding [13], laparoscopic surgery [14], groin-hernia repair [15], and lumbar-disc operations [16]. Video usage was also found to improve patient experience in cases of third-molar extractions [17] and for patients undergoing intravenous contrast for computed-tomography (CT) scans [18]. Although such studies have found that patients reported high satisfaction with the video and felt it provided necessary surgical information, there was evidence that the video might actually increase anxiety and worsen patient experience; instances when videos did not positively affect patient satisfaction have been observed in patients undergoing back surgery and total knee replacement [19, 20]. However, in the knee replacement study, such assessments came long after the informed consent process, in contrast to the present study's immediate test; administering a satisfaction survey during a three-month follow-up visit entails waiting far too long to assess a patient's satisfaction with the consent process [20]. Meanwhile, for the back surgery patients, researchers did record positive effects on the day of surgery (as did the present study) but not at later dates (e.g., a week after surgery) [19]. It seems more appropriate for patient satisfaction with the consent process to be evaluated shortly after surgery, preferably on the same day. Patient comprehension of informational videos has been studied previously in relation to cataract surgery [7, 9], but no study has focused on patient satisfaction.

Several possible reasons can explain why using an informational video leaded to beneficial outcomes. First, videos provide comprehensive information without the need to rely on literacy and preferred languages to reach separate patient populations. It has been demonstrated that videos tailored specifically to certain groups can enhance the effectiveness of multimedia during the informed consent process if linguistic or cultural barriers exist [18]. Second, this study emphasized the secondary role of the video since face-to-face discussions will always remain of primary importance to both surgeons and patients. The video in this study was simplified and focused on presenting the surgical procedure, the factors affecting postoperative visual improvement, and the overall risk of complications but left further discussion of each specific complication and risk to the patient's physician. Some authors have found that increased patient anxiety is associated with extensive presentation of a procedure's possible complications [21, 22] and that receiving too much information may actually prevent a patient from making a well-reasoned decision [23, 24]. Individual discussions of specific concerns and needs should therefore be conducted through face-to-face counseling. Furthermore, videos also have the advantage of allowing patients to rewatch segments repeatedly when necessary; in this study, repeated watching gave patients in the video group the confidence and knowledge needed for extensive discussions with their physicians [25].

Of course, although the standardized information delivery of a video improved patient satisfaction, it did not guarantee a better patient comprehension of the information presented. Possible explanations might include a relatively high level of understanding in the control patients and the very advanced ages and low education levels of the patients in general. It is an excellent performance by most standards that the control patients correctly answered questions on key aspects of cataract surgery 77–80% of the time. Although cataract surgery has been shown to be safe even among the very elderly population, old age and low education level did have a direct negative effect on patient comprehension and recall of the information presented to them [2]. This study's findings also agree with those of previous studies that showed that informational videos may not consistently result in improved patient comprehension over traditional oral counseling processes [8, 26, 27], especially with illiterate patients [26, 27]. Nevertheless, patients cannot meaningfully participate in making decisions about their medical care without adequately understanding the procedures' risks, benefits, and alternatives. Although the sampled patients correctly answered questions on key aspects of cataract surgery 77–80% of the time (an excellent performance by most standards), researchers should aim for even better results; more effort is needed to optimize the video's informative value and increase patients' abilities to remember the facts about the procedure and its risks, while also maintaining patient satisfaction.

The present study had certain limitations. The first was that this study was not performed in a double-blind research design, which might have especially affected the patients' informed consent discussions, creating bias and variation effects for the time the ophthalmologists spent on the consent process since a patient's knowledge after watching the informational video was obvious to staff members. However, data collection and analysis was carried out by a researcher who did not participate in any other parts of the trial. Meanwhile, the second major limitation of this study was that the questionnaire about the benefits and risks of cataract surgery had not been validated. It consisted of 10 simple yes-or-no questions that might not have accurately reflected the over-all information and cannot exclude the possibility that the yes-or-no questions did not capture the better understanding of the study group. The third limitation was that this study did not take into account patient preferences regarding the informed consent process. Some patients may prefer not to know the details of the risks of the procedure. Other limitations might include no replayed times of the video recorded in the study group and the unknown influence of background information about the sampled patients.

In summary, the present study shows that adding video assistance to traditional verbal informed consent advisement positively affected the patient journey, as is evident from the high satisfaction scores of the patients and the less required time with their ophthalmologists. This might have significant implications for improving the overall quality of patient care. Further efforts are needed to optimize the information presented by the video so as to improve patient comprehension.

## Figures and Tables

**Table 1:** Baseline characteristics of patients randomly selected for each group.

Characteristics	Video group (*n* = 40)	Control group (*n* = 40)
Age distribution		
50–70, years (%)	29 (73)	27 (67)
71–90, years (%)	11 (27)	13 (33)
Sex, female/male (%)	19/21 (48/52)	22/18 (55/45)
Preferred language		
Mandarin (%)	6 (15)	10 (25)
Cantonese (%)	34 (85)	30 (75)
Highest education level		
Illiterate (%)	5 (13)	4 (10)
Primary education (%)	27 (67)	26 (65)
Secondary or higher	8 (20)	10 (25)
education (%)		
Computer use		
Rarely/never (%)	29 (73)	30 (75)
Often (%)	11 (27)	10 (25)

**Table 2:** Patient satisfaction survey on the informed-consent process.

Items	Number of very satisfied or satisfied patients (video group/control group)	*p*
Patient was invited to ask questions (%)	38/37 (95/93)	0.500
Attitude of ophthalmologist while speaking with patient (%)	35/31 (88/78)	0.189
Patient received enough informed-consent counseling prior to cataract surgery (%)	39/34 (98/85)	0.054
Patient was given enough time to understand the procedure before signing the informed-consent form (%)	31/24 (78/85)	0.074
Overall satisfaction with the informed-consent process (%)	34/26 (85/65)	0.035

**Table 3:** The influence of age distribution, gender, preferred language, highest education level, and computer use on the number of correct responses out of the 10-item questionnaire (accuracy rate) in each group.

Characteristics	Video group	Control group
Age distribution		
50–70 years	246 (84%)	243 (91%)
71–90 years	75 (69%)	67 (51%)
Sex, female/male (%)		
Female	146 (76%)	159 (72%)
Male	175 (84%)	151 (84%)
Preferred language		
Mandarin	44 (74%)	70 (70%)
Cantonese	277 (81%)	240 (80%)
Highest education level		
Illiterate	37 (71%)	28 (70%)
Primary	206 (77%)	195 (75%)
Secondary or higher	78 (97%)	87 (87%)
Computer use		
Rarely/never	213 (73%)	223 (74%)
Often	108 (99%)	87 (87%)

All *p* > 0.05.
